# Physical and chemical stability of Taxotere^®^ (docetaxel) one-vial (20 mg/ml) infusion solution following refrigerated storage

**DOI:** 10.3332/ecancer.2010.202

**Published:** 2010-12-15

**Authors:** M Hart, S Acott

**Affiliations:** 1 Analytical Development; 2 Analytical Development Leader, Industrial Affairs, sanofi-aventis, Rainham Road South, Dagenham, Essex RM10 7XS, UK

## Abstract

A one-vial formulation of the chemotherapy agent Taxotere^®^ (docetaxel) is likely to be commercially available in some countries in 2010. Through control of two significant potential risk factors for crystallization—intervention into the infusion bag (by using a one-step technique) and temperature (by use of refrigerated storage)—it was postulated that the risk of precipitation would be reduced and the storage time of Taxotere^®^ increased using the one-vial formulation versus the two-vial formulation. Furthermore, improved convenience and flexibility were anticipated as a result of the easier and quicker preparation associated with the one-vial formulation versus the two-vial formulation. The results of the physicochemical stability study presented here indicate that the one-vial formulation is indeed associated with reduced risk of precipitation of docetaxel. Moreover, refrigerated storage has been shown to extend the physical and chemical stability for up to seven days, versus 4 h for the currently approved two-vial formulation and 6 h for the one-vial formulation stored at room temperature. However, only physical and chemical stability have been assessed in this study.

## Introduction

Taxotere^®^ (sanofi-aventis, Bridgewater, NJ) is a potent chemotherapeutic agent approved for use in breast cancer, non-small-cell lung cancer, metastatic castrate-resistant prostate cancer, gastric adenocarcinoma and squamous cell carcinoma of the head and neck [1–3]. The active ingredient of Taxotere^®^ is docetaxel, which is a lipophilic molecule with a low-aqueous solubility. To allow administration of the drug by infusion, docetaxel is solubilized by micelle formation with polysorbate 80.

To achieve the required solubility, Taxotere^®^ has been packaged as a two-vial presentation since its introduction to the market in 1995. The two-vial presentation requires two dilutions prior to administration. The first vial (concentrate) contains docetaxel in polysorbate 80, at a concentration of 40 mg/ml. The second vial contains a 12% w/v ethanol/water diluent. When the entire extractable volume of the diluent vial is added to the concentrate vial and mixed, a 10 mg/ml intermediate (premix) solution of docetaxel is generated, which is then dispensed into an infusion solution containing 0.9% saline or 5% dextrose for administration to the patient. An infusion solution prepared with 2-vial Taxotere^®^, if stored at 2°C–25°C, is stable for 4 h according to the package information [1]. Data published by Thiesen and Kramer in 1999 suggested acceptable physicochemical stability for up to 28 days [4]; however, despite the increased potential for wastage and higher overall drug expenditures [5], the shorter 4-h expiry date is generally adhered to in practice because of a perceived lack of convincing experimental data in favour of the longer storage period and continued publication of conflicting data [6].

The main reason for the short storage time is that there is a potential risk of crystallization of the Taxotere^®^ infusion solution during storage. The factors potentiating this risk include: (a) insufficient mixing of the premix solution; (b) excessive agitation; (c) the presence of particles and surface irregularities; (d) physical interventions into the solution and (e) temperature.

Insufficient mixing of the premix solution can lead to gel phase formation, which is transferred to the infusion bag and, in turn, can lead to precipitation of docetaxel through competition with micelle formation. Excessive agitation can lead to generation of additional nucleation sites through bubble formation, as well as imparting energy into the system, which promotes the achievement of equilibrium conditions. Nucleation would be expected to be promoted by particles in the solution, as well as surface irregularities in the infusion bags. Physical interventions into the infusion solution, such as the introduction of a syringe needle into the solution, provide additional nucleation sites. Finally, temperature affects the kinetics of the release of docetaxel from the micelles in the supersaturated system. A lower temperature is expected to lead to stabilization of the micelles.

A one-vial formulation of Taxotere^®^ has recently been developed and is likely to be commercially available in 2010 in some countries. This formulation consists of a 20-mg/ml solution of docetaxel diluted in 50/50 v/v polysorbate 80/ethanol and maintains the same docetaxel/polysorbate ratio as in the two-vial preparation. Preparation of an infusion solution using this 1-vial Taxotere^®^ is simplified in that no premix solution is required and the appropriate dose of concentrate is simply added to the infusion bag, with gentle mixing (one-shot preparation technique). With no intermediate premix solution (which is itself a supersaturated micellar solution and can potentially crystallize) [7], the risk of crystallization should be reduced.

The aims of this study were to determine the potential storage time for Taxotere^®^ one-vial infusion bags under refrigerated conditions with respect to physical stability and to confirm chemical stability during the storage period.

*The results of the study were presented as a poster at the European Society of Clinical Pharmacy (ESCP) European Symposium between October 21 and 23, 2010* [8]*, and are discussed in more detail here.*

## Materials and methods

Taxotere^®^ one-vial infusion solutions were prepared by adding 200 mg of docetaxel, using a single introduction of 10 ml docetaxel concentrate (20 mg/ml) into 250 ml polyethylene bags filled with 0.9% saline. Infusion bags were stored under refrigeration at 5°C and removed after either two (*n*=60 bags) or seven (*n*=60 bags) days and assessed immediately and at intervals after removal and storage for a further 24 h at ambient temperature (about 20°C). Crystallization was assessed by a careful visual examination. The chemical stability was assessed by determination of the docetaxel content and degradation impurities in five of the infusion bags (three bags after refrigerated storage for two days and three bags after refrigerated storage for seven days) by high-performance liquid chromatography.

## Results

No visible crystallization was observed in any Taxotere^®^ one-vial infusion bags following refrigerated storage for either two or seven days and subsequent storage for 24 h at approximately 20°C. There was no significant evolution of pH, docetaxel content or degradation impurities in the infusion bags during this time.

## Discussion

Physical and chemical stability of a Taxotere^®^ infusion depends on minimizing the risk of crystallization during storage. Various factors potentiate this risk as discussed in the introduction.

This study sought to control two significant potential risk factors for crystallization—intervention into the infusion bag (by using a one-step technique) and temperature (by use of refrigerated storage). In this way, it appears possible to extend the storage time of Taxotere^®^ one-vial infusion bags from 4 h with the two-vial infusion preparation to seven days, leading to increased convenience and flexibility in the pharmacy and clinic when needed.

Our findings build on those published by Pinguet and colleagues, who noted no crystallization at preparation time and good physical stability at 24 h for infusion bags prepared using the one-vial formulation [9]. Their study also showed that the preparation time for 1-vial Taxotere^®^ was significantly shorter than that for 2-vial Taxotere^®^ and the amount of materials used for preparation significantly reduced [9].

## Conclusion

The new Taxotere^®^ one-vial formulation is associated with reduced risk of precipitation of docetaxel via elimination of the premix solution (required for the Taxotere^®^ two-vial presentation) and a one-shot preparation technique (i.e. only one intervention into the infusion bag). Moreover, refrigerated storage has been shown to extend the physicochemical stability of the formulation to up to seven days (versus 4 h with the previous formulation).
